# Death of M. Lisfranc

**Published:** 1847-07

**Authors:** 


*Death of M. Lisfranc.*—The celebrated surgeon of La Pitie died on the 12th of May from the progress of diphtheriticangina, complicated with pernicious fever, during the second paroxysm of which he expired. M. Lisfranc was only sixty years of age. The funeral took place on the 14th inst. It was most numerously attended, and MM. Pariset and Serres, and others, pronounced on the grave speeches expressive of their feelings on the melancholy occasion. As an operator, Lisfranc was unequalled, and it is a serious misfortune that his work on Operative Surgery, though far advanced, was not completed at the time of his death; his lectures, of the most practical nature, were always fully attended, and were remarkable at all times for the energy with which his opinions were expressed. Lisfranc has written few works: two volumes of clinical surgery, and an incomplete sketch of the art of operations; but in his books, as well as in his practice, the following principle is constantly illustrated:—"The operations of surgery are brilliant; but the art of the surgeon consists less in performing them with ability, than in rendering them useless by proper treatment." Lisfranc's researches on cancer, on uterine disease, on white swellings, and his method for the partial amputation of the foot, ensure to his name a place amongst those of the most distinguished surgeons of the French school.—*London Med. Times.*

